# Coexistence of Upper Airway Obstruction and Primary and Secondary Enuresis Nocturna in Children and the Effect of Surgical Treatment for the Resolution of Enuresis Nocturna

**DOI:** 10.1155/2014/656431

**Published:** 2014-08-20

**Authors:** Gül Soylu Özler, Serkan Özler

**Affiliations:** ^1^Department of Otorhinolaryngology, Mustafa Kemal University, Hatay 31100, Turkey; ^2^Clinic of Urology, Antakya Government Hospital, Hatay 31100, Turkey

## Abstract

*Objective.* The aim of this study is to investigate the coexistence of upper airway obstruction (UAO) and primary enuresis nocturna (PEN) and secondary enuresis nocturna (SEN) in children. Besides, the efficacy of surgery on resolution of enuresis nocturna is evaluated. *Materials and Methods.* The children with PEN and SEN were included in the first group and investigated for UAO in the Department of Otorhinolaryngology. During the same period, children who had been planned for an operation to treat UAO over 5 years old were included in the second group and were evaluated in the Department of Urology for PEN and SEN before the operation. *Results.* A hundred patients completed the study (50 patients in Group 1, 50 patients in Group 2). According to the otolaryngologic examination, 20 of 25 PEN patients and 9 of 25 SEN patients also had UAO. The difference was statistically different (*P* < 0.05). The second group consisted of fifty patients on the surgery list for upper airway obstructive pathologies. Coexistence of PEN and SEN is found in 12 and 3 of children, respectively. These ratios were statistically significant (*P* < 0.05). The improvement rate of PEN and SEN after operation in the second group was 83.3% and 33.3%, respectively. The difference was statistically significant (*P* < 0.05). *Conclusion.* There is a strong relationship between PEN and UAO, but it cannot be declared for SEN patients. UAO should be kept in mind as a possible etiologic factor for PEN.

## 1. Introduction 

Upper airway obstruction (UAO) is estimated to be seen at 27% of paediatric population [1]. Nasal and/or oropharyngeal pathologies may be the reason of UAO. The most common cause is adenotonsillar hypertrophy [2]. Enuresis nocturna (EN) also called as intermittent nocturnal incontinence means the involuntary and recurrent wetting episodes during sleep. EN has two types: primary type, in which the child has never been dry at night, and the secondary type, in which a child has been dry for 6 months or more and then begins to wet the bed [3]. EN is reported in 8–47% of children with UAO and the improvement rate of enuresis after surgery in these patients is up to 76% [4]. Although there are several studies investigating the relationship between EN and UAO, there is still a lack of detailed prospective studies. Moreover, most of the studies did not make a distinction between primary and secondary types of enuresis. The aim of this study is to investigate the coexistence of UAO and PEN and SEN in children. Besides, the efficacy of surgery on resolution of enuresis nocturna is evaluated.

## 2. Materials and Methods

This study was performed between September 2009 and May 2011 with the collaboration of the two departments (Otorhinolaryngology and Urology Clinics). During the study, the children with PEN and SEN were included in the first group and investigated for pathologies causing UAO in the Department of Otorhinolaryngology. A detailed history of obstructive symptoms, a routine otolaryngologic examination was performed, and, if indicated, nasal endoscopy and lateral cephalography were used. The patients with detected UAO pathologies in the first group underwent surgery to treat UAO. During the same period, children who had been planned for an operation to treat pathologies causing UAO over 5 years old were included in the second group and were evaluated in the Department of Urology for EN before the operation. Surgery decision was made according to the detailed history, physical examination, and lateral cephalometric examination. Indications for surgery for UAO were as follows: snoring and sleep apnea lasting at least 10 seconds, witnessed by the parents; hypertrophic tonsils detected by physical examination; obstructing adenoid tissue examined by nasal endoscopy or lateral cephalometric examination. Diagnostic criteria from the fourth edition of the Diagnostic and Statistical Manual for Mental Disorders (DSM-IV) were used for diagnosis of enuresis. DSM-IV diagnostic criteria for enuresis are as follows [5].

- Repeated voiding of urine into bed or clothes (involuntary or intentional).
- The behaviour is clinically significant as manifested by either a frequency of twice a week for at least 3 consecutive months or the presence of clinically significant distress or impairment in social, academic (occupational), or other important areas of functioning.
- Chronological age is at least 5 years (or equivalent developmental level).
- The behaviour is not due to the direct physiological effect of a substance (e.g., a diuretic) or a general medical condition (e.g., diabetes, spina bifida, and a seizure disorder).

Children with congenital anomalies, mental retardation, systemic diseases, central nervous system diseases, and any current medication were excluded.

The patients who underwent surgery for UAO in both groups were followed by telephone calls with the parents after the operation and the patients were followed for 3 to 12 months for the resolution of enuresis. If the patient was dry for at least 3 months, it was accepted as complete cure. At least 50% improvement in enuresis frequency in children was accepted as a partial response. Ethics committee approval was obtained and the study was conducted in adherence to the Declaration of Helsinki. Informed consent was obtained from the parents of participants.

### 2.1. Statistical Analysis

Statistical analysis was performed using the Statistical Package for the Social Sciences (SPSS) 13.0 Evaluation for Windows. Chi-square test was used for nominal variables. Statistically significant level was accepted as *P*  value < 0.05.

## 3. Results

A hundred patients completed the study (50 patients in Group 1, 50 patients in Group 2). Fifty patients were included in the first group and the ages were between 6 and 17 years (8.5 ± 2.71). There were 31 male and 19 female patients in this group. 25 patients were PEN; 25 patients were SEN. Marked septal deviation in 3 patients (16%), moderate septal deviation in 2 patients (12%), adenoid hypertrophy in 5 patients (20%), adenotonsillar hypertrophy in 5 patients (36%), inferior turbinate hypertrophy in 4 patients (20%), and antrochoanal polyp in 1 patient (4%) were examined in PEN patients. Marked septal deviation in 1 patient (4%), moderate septal deviation in 1 patient (4%), adenoid hypertrophy in 2 patients (8%), adenotonsillar hypertrophy in 2 patients (8%), and inferior turbinate hypertrophy in 3 patients (12%) were examined in SEN patients (Table 1). According to the otolaryngologic examination, 20 of 25 PEN patients (80%) and 9 of 25 SEN patients (36%) also had UAO. The difference was statistically significant (*P* < 0.05).

The second group consisted of fifty patients on the surgery list for upper airway obstructive pathologies. The patients were between the ages 5 and 17 (8.54 ± 2.70). There were 29 male and 21 female patients. Coexistence of PEN and SEN is found in 12 (24%) and 3 (6%) of children, respectively. The coexistence ratios were statistically different (*P* < 0.05).

In the first group, 20 PEN patients and 9 SEN patients underwent surgery for UAO. Surgery for UAO was successful in follow-up. According to the telephone calls, 10 (50%) patients were completely cured, 6 (30%) had a partial response, and 4 (20%) of 20 patients had no response to surgical treatment in PEN group. In SEN group, 3 (33.3%) of 9 patients were completely cured, and 6 (66.6%) had no response. The improvement rates of PEN and SEN groups were 80% and 33.3%, respectively. The improvement rates were statistically significant (*P* < 0.05).

In the second group, adenoidectomy and/or adenotonsillectomy were performed in 45 cases and septoplasty was performed in 4 cases; endoscopic resection of antrochoanal polyp was performed in 1 case. According to the telephone calls with their parents, 6 (50%) patients were completely cured, 4 (33.3%) had a partial response, and 2 (15.1%) of 12 patients had no response to surgical treatment in patients with PEN. In SEN patients, no patient was completely cured, 2 (83.3%) had a partial response, and 1 (33.3%) of 3 patients had no response to surgical treatment (Table 2). The improvement rate of PEN and SEN after operation in the second group was 83.3% and 33.3%, respectively. The improvement rates were statistically significant (*P* < 0.05).

## 4. Discussion

EN is a frequent chronic illness in the pediatric population [4]. Previous studies reported its frequency between 5 and 15% [6, 7]. Etiology of EN is still controversial. Delay of nervous system maturation, low bladder capacity, abnormalities of the urinary tract, inadequate secretion of antidiuretic hormone (ADH), genetic factors, immature waking mechanisms, deep sleep, neurologic bladder problems, bacteriuria, diet, socioeconomic status, and psychogenic factors were suggested as etiologic factors [3, 4]. Aydil et al. reported that every two of three PEN patients (65.6%) had some form of UAO [8]. In our study, the otorhinolaryngologic examination of children with enuresis nocturna revealed that 80% of PEN patients and 36% of SEN patients had UAO. Our results for PEN are in agreement with the literature. We could not compare the results for SEN, because we could not find any data in the literature for SEN patients.

According to Brooks and Topol, children with more than one respiratory event per hour of sleep are at a significantly greater risk of enuresis than are children with a respiratory event less than one [4]. The rate of EN in children with UAO has been reported to be between 20 and 34.5% in previous studies [3, 7, 8]. In our study, the rate of PEN in children with UAO was 24%, whereas it was 6% for SEN. The results for PEN are consistent with the literature. We could not compare the results for SEN, because we could not find any data in the literature for SEN patients.

If EN is seen more frequently in patients with UAO, the surgery for UAO may be a treatment option for EN. The cure rate of EN after surgery is reported to be 62–90 % in the literature [3, 7, 8]. The improvement rate of PEN and SEN after surgery was 83.3% and 33.3%, respectively. These results were similar to previous studies.

There are several hypotheses to explain the relationship between the UAO and the EN. Brooks and Topol believe that UAO have negative effects on arousal response [6]. Maddern suggested that EN is a result of decreased neuromuscular tonus during sleep which is more significant in patients with UAO [9]. Yeung et al. suggested that temporary fall in oxygen saturation in UAO patients leads to loss of bladder control. They indicated that a number of patients who could not be aroused before surgery were able to wake up and go to the bathroom after surgery [10].

Besides this, upper airway resistance causes high inspiratory effort, and this effort results in high intrathoracic negative pressure that leads to cardiac distention, natriuretic response, and atrial natriuretic peptide (ANP) secretion [2] As a result, ANP enhances natrium/water excretion and inhibits other hormones, which regulates fluid balance and the renin-angiotensin system.

Weider et al. found that the diurnal ADH rhythm disappeared in these patients [11]. In fact, dysrhythmic ADH secretion is a result of an atrial natriuretic response to upper airway resistance. Norgaard and Djurhuus found that patients with EN have lacked the nocturnal increase in ADH levels and had nocturnal urine production up to four times the volume of functional bladder capacity which explains spontaneous bladder emptying [12]. Contrary to this, Läckgren et al. did not find any difference in the diurnal ADH secretion between enuretic and normal controls [13].

Although there are previous studies in the literature that investigated the relationship between UAO and EN, this paper is the first study that made a distinction of PEN and SEN. Limitations of this study is that we did not perform polysomnography for the UAO patients. However it is an expensive test and it is still controversial whether it is necessary to perform polysomnography as a routine preoperative test to document the presence of UAO in children with an obvious clinical situation [14].

## 5. Conclusion

In our opinion there is a strong relationship between PEN and UAO. UAO may be one of the etiologic factors in PEN. Children with PEN should be examined by an otorhinolaryngologist before treatment, because surgical treatment of UAO may be enough for total cure. But it cannot be declared for SEN patients. Nevertheless UAO should be kept in mind as a possible etiologic factor.

## Figures and Tables

**Table 1:** Otorhinolaryngologic examination of PEN and SEN patients.

	PEN (*n* = 25)	SEN (*n* = 25)
Marked septal deviation	3 (12%)	1 (4%)
Moderate septal deviation	2 (8%)	1 (4%)
Adenoid hypertrophy	5 (20%)	2 (8%)
Adenotonsillar hypertrophy	5 (20%)	2 (8%)
Inferior turbinate hypertrophy	4 (16%)	3 (12%)
Antrochoanal polyp	1 (4%)	0 (0%)

**Table 2:** The improvement rates after surgery in PEN and SEN patients.

	PEN (*n* = 12)	SEN (*n* = 3)
Complete cure	6 (50%)	0 (0%)
Partial response	4 (33.3%)	2 (83.3%)
No response	2 (15.1%)	1 (33.3%)
